# S_N_2 Reactions with an Ambident Nucleophile: A Benchmark Ab Initio Study of the CN^–^ + CH_3_Y [Y = F, Cl, Br, and I] Systems

**DOI:** 10.1021/acs.jpca.1c10448

**Published:** 2022-02-02

**Authors:** Zsolt Kerekes, Domonkos A. Tasi, Gábor Czakó

**Affiliations:** MTA-SZTE Lendület Computational Reaction Dynamics Research Group, Interdisciplinary Excellence Centre and Department of Physical Chemistry and Materials Science, Institute of Chemistry, University of Szeged, Rerrich Béla tér 1, Szeged H-6720, Hungary

## Abstract

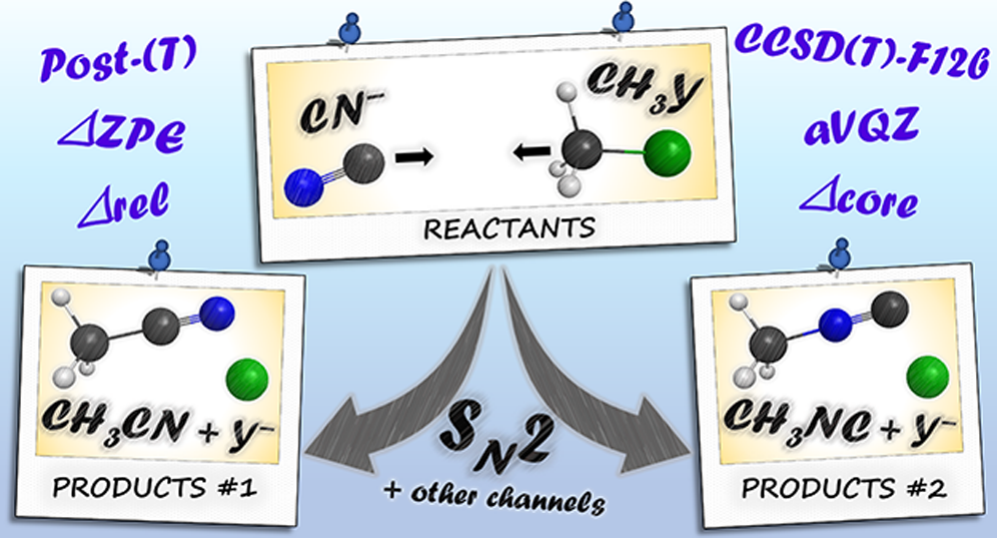

We characterize the Walden-inversion, front-side attack, and double-inversion S_N_2 pathways leading to Y^–^ + CH_3_CN/CH_3_NC and the product channels of proton abstraction (HCN/HNC + CH_2_Y^–^), hydride-ion substitution (H^–^ + YH_2_CCN/YH_2_CNC), halogen abstraction (YCN^–^/YNC^–^ + CH_3_ and YCN/YNC + CH_3_^–^), and YHCN^–^/YHNC^–^ complex formation (YHCN^–^/YHNC^–^ + ^1^CH_2_) of the CN^–^ + CH_3_Y [Y = F, Cl, Br, and I] reactions. Benchmark structures and frequencies are computed at the CCSD(T)-F12b/aug-cc-pVTZ level of theory, and a composite approach is employed to obtain relative energies with sub-chemical accuracy considering (a) basis-set effects up to aug-cc-pVQZ, (b) post-CCSD(T) correlation up to CCSDT(Q), (c) core correlation, (d) relativistic effects, and (e) zero-point energy corrections. C–C bond formation is both thermodynamically and kinetically more preferred than N–C bond formation, though the kinetic preference is less significant. Walden inversion proceeds via low or submerged barriers (12.1/17.9(F), 0.0/4.3(Cl), −3.9/0.1(Br), and −5.8/–1.8(I) kcal/mol for C–C/N–C bond formation), front-side attack and double inversion have high barriers (30–64 kcal/mol), the latter is the lower-energy retention pathway, and the non-S_N_2 electronic ground-state product channels are endothermic (Δ*H*_0_ = 31–92 kcal/mol).

## 1 Introduction

Bimolecular nucleophilic substitution (S_N_2) reactions have been widely studied both experimentally and theoretically, and their Walden-inversion and front-side attack mechanisms have been known at the atomic level since the 1930s.^1−6^ The traditional S_N_2 reaction pathway at the carbon center goes through pre- and post-reaction ion-dipole wells separated by a penta-covalent, usually submerged/high-energy transition state, where the angle of the nucleophile–central atom–leaving group is around 180°/90° for Walden inversion/front-side attack.^3,5^ However, recent works in the past two decades uncovered that the S_N_2 reactions are not so simple.^7−15^ Besides ion-dipole complexes, hydrogen- and halogen-bonded complexes can be formed in the entrance and/or exit channels, which may strongly affect the dynamics of the S_N_2 reactions.^10−15^ Moreover, post-reaction hydrogen-bonded complex formation may open new product channels for ion–molecule reactions, as recent dynamics studies showed in the case of the XH^–^ + CH_3_F [X = O and S] systems, which can lead to HF + CH_3_X^–^ products besides F^–^ + CH_3_XH.^15,16^ Furthermore, our dynamics simulations revealed a double-inversion mechanism for S_N_2 reactions, where a proton-abstraction induced inversion (first inversion) is followed by a substitution via the Walden-inversion transition state (second inversion), resulting in retention of the initial configuration.^9^

In the simplest S_N_2 reactions, the nucleophile is a halide or hydroxyl ion and most of the nontraditional S_N_2 pathways were uncovered by studying their reactions with methyl-halides.^4−6^ In the present study, we investigate the reactions of the simplest ambident nucleophile, the cyanide ion (CN^–^), with the CH_3_Y [Y = F, Cl, Br, and I] molecules. Ambident nucleophiles have two reactive centers like CN^–^, where the negative charge is delocalized, allowing the formation of C–C and C–N bonds in the S_N_2 reactions with CH_3_Y. Following the early experimental and theoretical investigations on the CN^–^ + CH_3_Y S_N_2 reactions,^17−21^ in 2003, Gonzalez et al.^22^ characterized the pre- and post-reaction ion-dipole complexes and the Walden-inversion transition state of the CN^–^ + CH_3_F system using the focal-point analysis approach based on MP2/aug-cc-pV5Z and CCSD(T)/aug-cc-pVTZ energies as well as considering core correlation and relativistic effects at the CCSD(T)/TZ2P + dif geometries. In 2014 and 2015, Wang and co-workers^23,24^ performed QM/MM computations in aqueous solution for the CN^–^ + CH_3_Br and CN^–^ + CH_3_Cl reactions, respectively. However, none of the abovementioned theoretical studies considered the ambident character of the CN^–^ nucleophile and only the thermodynamically favored C–C bond formation was investigated. In the early 2010s, Bierbaum and co-workers^25,26^ measured the rate coefficients and kinetic isotope effects for the CN^–^ + CH_3_I/CD_3_I systems using flowing afterglow-selected ion flow tube mass spectrometry, however, it was without distinguishing between the C–C and C–N bond formations. The first combined experimental–theoretical study on the CN^–^ + CH_3_I two-channel reaction was reported in 2015 by Wester and co-workers,^27^ where both the I^–^ + CH_3_CN/CH_3_NC S_N_2 Walden-inversion pathways were characterized using the CCSD(T)/aug-cc-pVTZ//MP2/aug-cc-pVDZ level of theory and velocity map imaging. Experimentally, the I^–^ anion was detected, and thus direct separation of the two different product channels was not possible. Nevertheless, the measured translational energy of I^–^ could be used to predict the neutral counterpart, allowing the experimental determination of the isomer branching ratios. In 2019, in our group, the reaction pathways of the CN^–^ + CH_3_Y [Y = F, Cl, Br, and I] systems were characterized using the explicitly correlated CCSD(T)-F12b/aug-cc-pV*n*Z [*n* = D, T, and Q] levels of theory.^14^ In the above study, for the first time, we considered front-side attack and double inversion for the CN^–^ nucleophile; however, we only investigated the C–C bond formations. In the present work, we report stationary points characterizing the C–N bond formations as well and we consider electron correlation beyond CCSD(T), core correlation, and scalar relativistic effects, thereby determining the benchmark energetics of the title reactions superseding the accuracy of previous work. Furthermore, besides the S_N_2 pathways, we compute the enthalpies of several additional product channels obtained by, for example, proton abstraction, halogen abstraction, and hydrogen substitution, considering the ambident character of the CN^–^ reactant, thereby anchoring the different asymptotes of the global potential energy surfaces (PESs) of the title reactions, by which information may be utilized in future analytical PES developments and reaction dynamics studies. In Section 2, we describe the computational details, the results are presented and discussed in Section 3, and the paper ends with summary and conclusions in Section 4.

## 2 Computational Details

The mapping of the stationary points for the title reactions is performed based on previous studies^14,21,22,27^ of the C–C bond-forming NC^–^ + CH_3_Y [Y = F, Cl, Br, and I] processes and chemical intuition. Initially, the structures are determined using the second-order Møller–Plesset perturbation theory (MP2)^28^ with the aug-cc-pVDZ basis set.^29^ To get the most accurate geometries, we use the explicitly correlated coupled-cluster singles, doubles, and perturbative triples method (CCSD(T)-F12b)^30^ with the correlation-consistent aug-cc-pVDZ and aug-cc-pVTZ basis sets. Harmonic vibrational frequencies are also calculated using the previously mentioned levels of theory. For the open-shell products, we use restricted second-order Møller–Plesset perturbation theory (RMP2)^31^ and the restricted open-shell Hartree–Fock-based unrestricted explicitly correlated coupled-cluster singles, doubles, and perturbative triples method (UCCSD(T)-F12b).^32^ For bromine and iodine, we employ a relativistic effective core potential (ECP), which replaces the inner-core 1s^2^2s^2^2p^6^ and 1s^2^2s^2^2p^6^3s^2^3p^6^3d^10^ electrons, respectively, and use the corresponding aug-cc-pV*n*Z-PP [*n* = D, T, and Q] basis sets.^33^ For the F12b computations, the default auxiliary basis sets are used as implemented in Molpro.^34^

To achieve sub-chemical accuracy, the following single-point energy computations are also performed at geometries obtained at the CCSD(T)-F12b/aug-cc-pVTZ level of theory:

(1) CCSD(T)-F12b/aug-cc-pVQZ to account for basis set effects.
(2) Coupled-cluster, singles, doubles, and triples [CCSDT]^35^ and coupled-cluster, singles, doubles, triples, and perturbative quadruples [CCSDT(Q)]^36^ methods with aug-cc-pVDZ basis to calculate post-CCSD(T) correlation. The corrections are defined as follows:

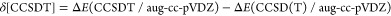
1
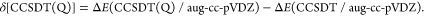
2

(3) The CCSD(T) method with the aug-cc-pwCVTZ basis^37^ is used to calculate frozen-core (FC) and all-electron (AE) energies. The core correction is as follows:

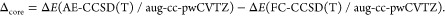
3

As default, the frozen-core approach correlates only the valence electrons, while the all-electron method correlates both valence electrons and the outer-core electrons on the main shell below the valence shell. For example, in the case of Y = F, Cl, Br, and I, all-electron means 1s^2^2s^2^2p^5^, 2s^2^2p^6^3s^2^3p^5^, 3s^2^3p^6^3d^10^4s^2^4p^5^, and 4s^2^4p^6^4d^10^5s^2^5p^5^, respectively.

(4) Douglas–Kroll (DK)^38^ AE-CCSD(T) computations are performed with the DK-optimized aug-cc-pwCVTZ-DK basis set^39^ to determine the scalar relativistic effects in case of Y = F and Cl. The relativistic correction can be obtained as

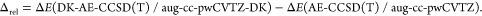
4

We are not able to determine the scalar relativistic effect for Y = Br and I via eq 4 because non-DK computations have to use ECPs with the PP basis sets for Br and I, which already incorporate scalar relativistic effects for these atoms. In order to estimate the uncertainty of the ECP computations, we compare the DK-AE-CCSD(T)/aug-cc-pwCVTZ-DK (without ECP) and AE-CCSD(T)/aug-cc-pwCVTZ-PP (with ECP) energies in the case of Y = Br and I. However, these energy differences are not included in the final benchmark data.

The following expression is used to calculate the benchmark classical relative energies for the CN^–^ + CH_3_Y [Y = F and Cl] systems:
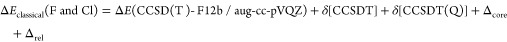
5and for Y = Br and I
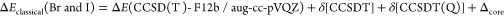
6where classical refers to static nuclei without zero-point energy (ZPE). We can compute the adiabatic benchmark energies with the following equations:
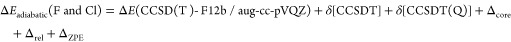
7and
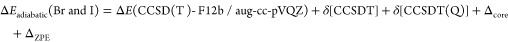
8where Δ_ZPE_ is the harmonic zero-point energy correction obtained at the CCSD(T)-F12b/aug-cc-pVTZ level of theory.

Computations up to CCSD(T) and CCSD(T)-F12b are performed with the Molpro^34^ ab initio program package. CCSDT and CCSDT(Q) energies are obtained with Mrcc^40,41^ interfaced to Molpro.

## 3 Results and Discussion

**Figure 1:**
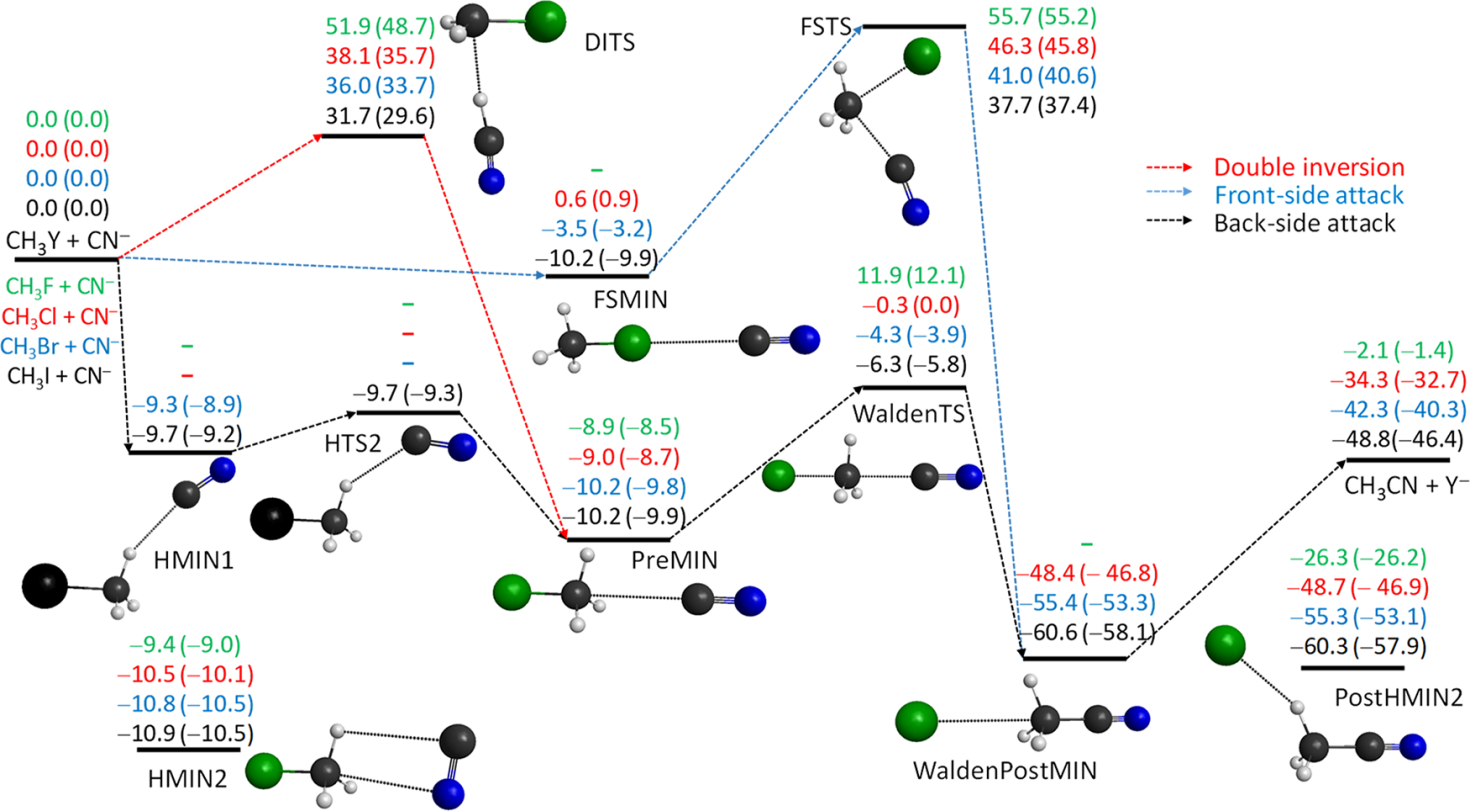
Benchmark classical (adiabatic) relative energies, in kcal/mol, of the stationary points along the different reaction pathways of the NC^–^ + CH_3_Y [Y = F, Cl, Br, and I] C–C-bond-forming S_N_2 reactions. The benchmark relative energies are obtained as CCSD(T)-F12b/aug-cc-pVQZ(-PP for Y = Br and I) + δ[T] + δ[(Q)] + Δ_core_ (+ Δ_rel_ for Y = F and Cl) (+ Δ_ZPE_ for adiabatic).

**Figure 2:**
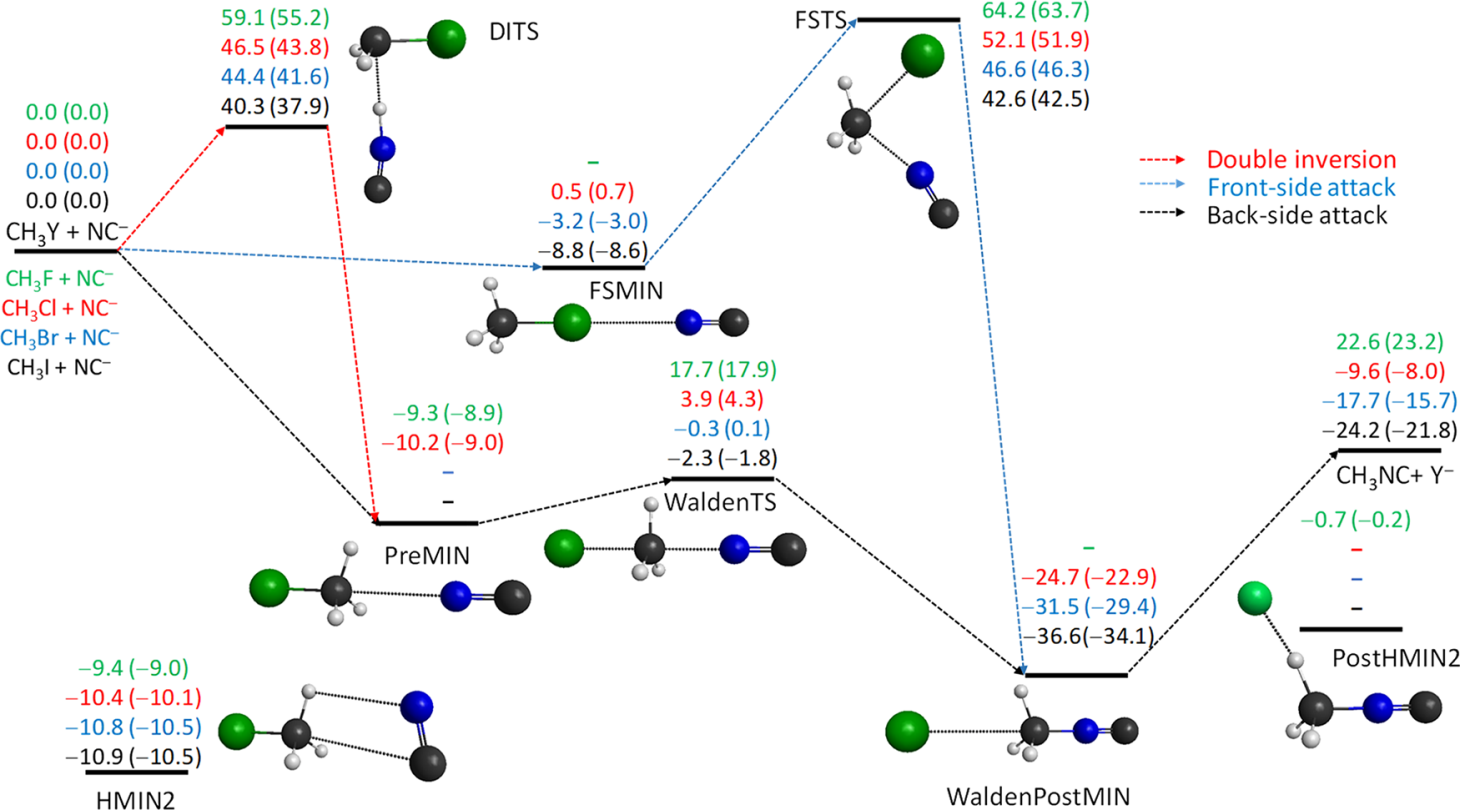
Benchmark classical (adiabatic) relative energies, in kcal/mol, of the stationary points along the different reaction pathways of the CN^–^ + CH_3_Y [Y = F, Cl, Br, and I] N–C-bond-forming S_N_2 reactions. The benchmark relative energies are obtained as CCSD(T)-F12b/aug-cc-pVQZ(-PP for Y = Br and I) + δ[T] + δ[(Q)] + Δ_core_ (+ Δ_rel_ for Y = F and Cl) (+ Δ_ZPE_ for adiabatic).

**Figure 3:**
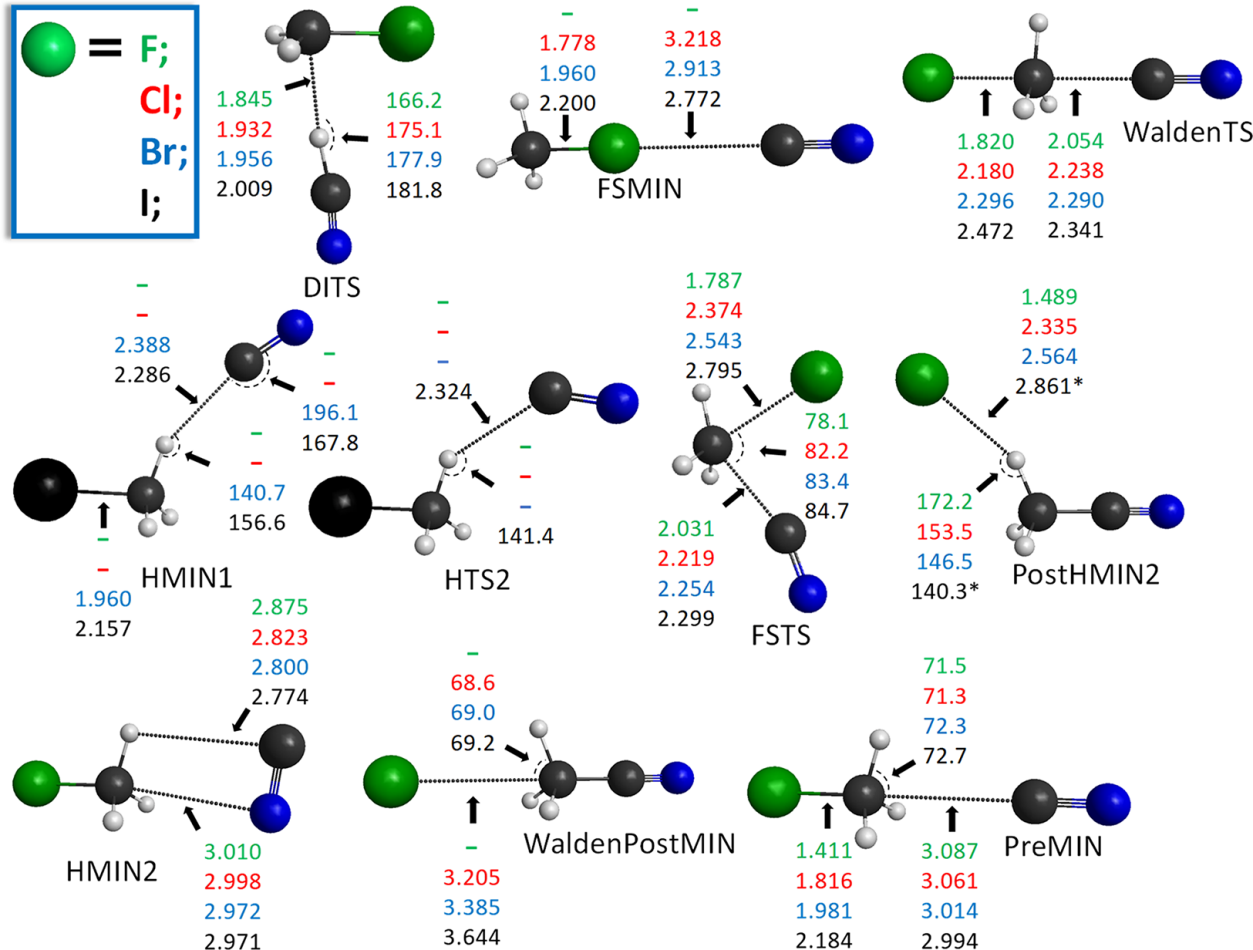
Benchmark structures of the stationary points for the NC^–^ + CH_3_Y [Y = F, Cl, Br, and I] C–C-bond-forming S_N_2 reactions showing the most important distances (Å) and angles (°) obtained at the CCSD(T)-F12b/aug-cc-pVTZ level of theory. The asterisk denotes MP2/aug-cc-pVDZ data.

**Figure 4:**
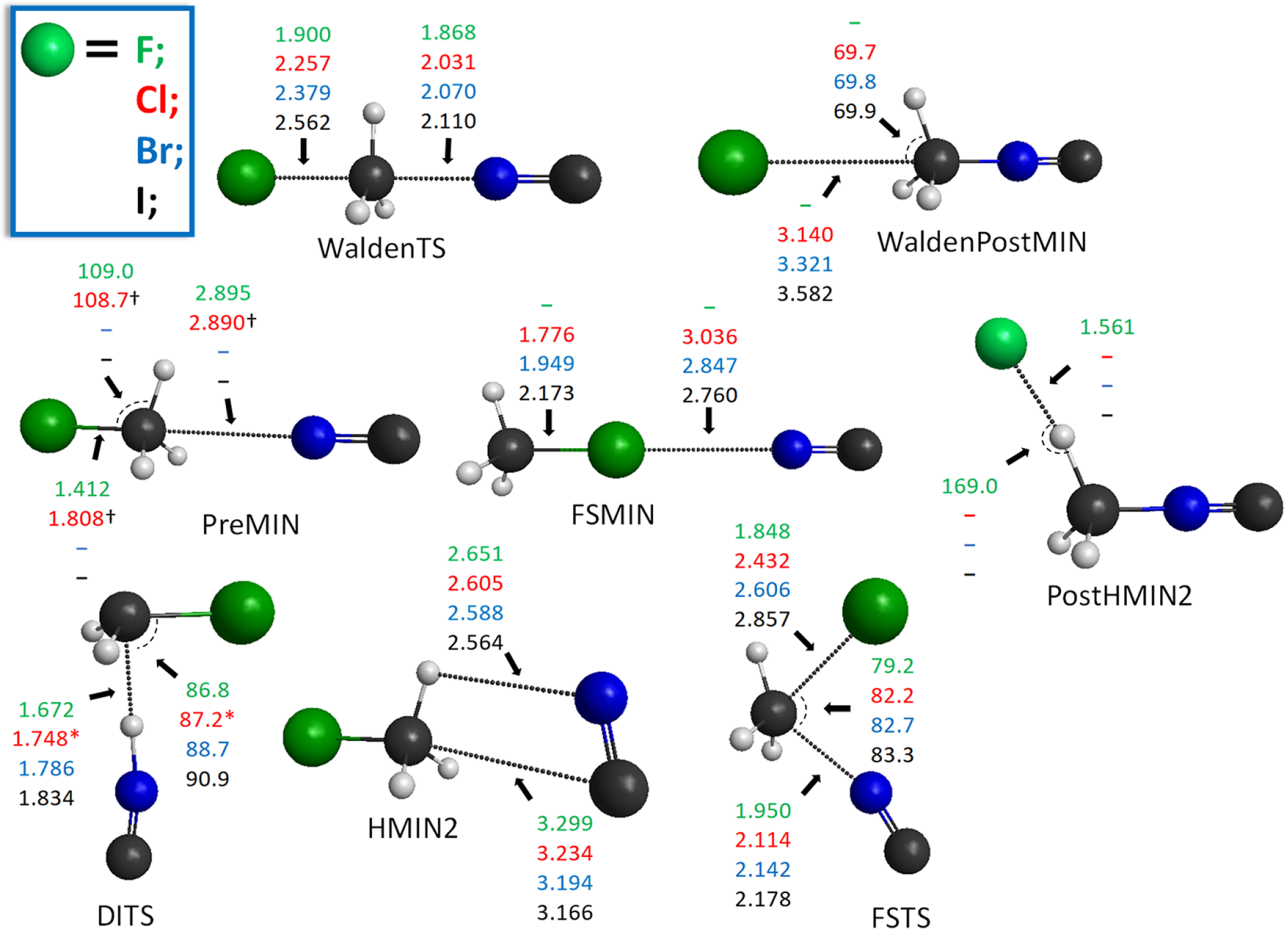
Benchmark structures of the stationary points for the CN^–^ + CH_3_Y [Y = F, Cl, Br, and I] N–C-bond-forming S_N_2 reactions showing the most important distances (Å) and angles (°) obtained at the CCSD(T)-F12b/aug-cc-pVTZ level of theory. The asterisk and dagger symbol denote MP2/aug-cc-pVDZ and CCSD(T)-F12b/aug-cc-pVDZ data, respectively.

Schematic potential energy surfaces of the NC^–^/CN^–^ + CH_3_Y [Y = F, Cl, Br, and I] C–C/N–C bond-forming S_N_2 reactions showing the benchmark stationary-point relative energies along the back-side attack (Walden-inversion), front-side attack, and double-inversion pathways are given in Figures 1 and 2, respectively. The geometries of the S_N_2 stationary points highlighting the most important benchmark structural parameters are shown in Figures 3 and 4 for the C–C and N–C bond formations, respectively. Qualitatively the C–C and N–C bond-forming S_N_2 reactions proceed via similar pathways, though subtle differences exist. Back-side attack Walden inversion goes through a *C*_3v_ central transition state (WaldenTS) and forms the products via a deep minimum (WaldenPostMIN, *C*_3v_) along collinear N/C–C–Y arrangement, except for Y = F, where only a hydrogen-bonded minimum (PostHMIN2, *C*_s_) is found in the exit channel. In the case of the C–C-bond-forming S_N_2 channel, PostHMIN2s exist for Y = Cl, Br, and I as well, and their energies are similar to those of the corresponding WaldenPostMINs. However, PostHMIN2 with the N–C bond has only been found for Y = F. In the entrance channel, more differences are observed depending on the reactive center of the ambident nucleophile. Ion-dipole complexes (PreMIN) with *C*_3v_ point-group symmetry are formed for all Y if NC^–^ reacts with its C-side, whereas PreMIN is only obtained for Y = F and Cl in the case of N–C bond formation. Hydrogen-bonded complexes (HMIN1) with nearly collinear H···CN are obtained only for H···C bonding and Y = Br and I; however, HMIN1 complexes are slightly less stable than PreMINs. For Y = I, a transition state (HTS2) connecting HMIN1 and PreMIN is also found. In all the NC^–^/CN^–^ + CH_3_Y [Y = F, Cl, Br, and I] cases, a non-traditional complex (HMIN2) also exists in the entrance channel, which corresponds to the deepest minimum in the pre-reaction well. For Y = Cl, Br, and I, halogen-bonded minima (FSMIN, *C*_3v_) are found for both Y···CN and Y···NC bonding, which are unbound for Y = Cl and the most stable for Y = I. The front-side attack retention pathways go over a high-energy transition state (FSTS) with Y–C–C/N angles around 80°. Double inversion opens a slightly lower-energy retention pathway, where the first inversion occurs via a so-called double-inversion transition state (DITS), having a nearly collinear C···HCN or C···HNC arrangement. This first, proton-abstraction-induced inversion is followed by a substitution via WaldenTS, resulting in retention of the initial configuration. Quantitatively, the main difference between the NC^–^/CN^–^ + CH_3_Y reactions is that thermodynamically, the Y^–^ + CH_3_CN formation is clearly favored over the Y^–^ + CH_3_NC channel, as the latter is above the former by 24.6 kcal/mol. The C–C bond-forming S_N_2 reactions are exothermic with 0 K reaction enthalpies ranging from −1.4 (Y = F) to −46.4 (Y = I) kcal/mol, whereas in the case of N–C bond formation, the S_N_2 channel is endothermic for Y = F (Δ*H*_0_ = 23.2 kcal/mol) and exothermic, Δ*H*_0_ = −8.0, −15.7, and −21.8 kcal/mol, for Y = Cl, Br, and I, respectively. The dissociation energies of the WaldenPostMINs are similar for the Y^–^···H_3_CCN and Y^–^···H_3_CNC complexes, i.e., around 12–15 kcal/mol with only slight Y dependence. The energies of the WaldenPostMINs relative to the reactants are of course deeper by about 24 kcal/mol for the former, similar to the reaction enthalpies. In the entrance channel, significant energy differences are not found for the C–C and N–C bonded complexes. At the transition states (WaldenTS, FSTS, and DITS), C–C bond formation is energetically preferred by about 4–9 kcal/mol relative to N–C bonding. The classical barrier heights for C–C bond formation via Walden inversion are 11.9, −0.3, −4.3, and −6.3 kcal/mol for Y = F, Cl, Br, and I, respectively, whereas the corresponding values are 17.7, 3.9, −0.3, and −2.3 kcal/mol for N–C bond formation. The FSTSs are in the energy ranges of 55.7–37.7 (C–C) and 64.2–42.6 (N–C) kcal/mol, whereas the DITSs are 51.9–31.7 and 59.1–40.3 kcal/mol, in order. Thus, we can conclude that the thermodynamically strongly favored C–C formation is kinetically only slightly preferred.

**Figure 5:**
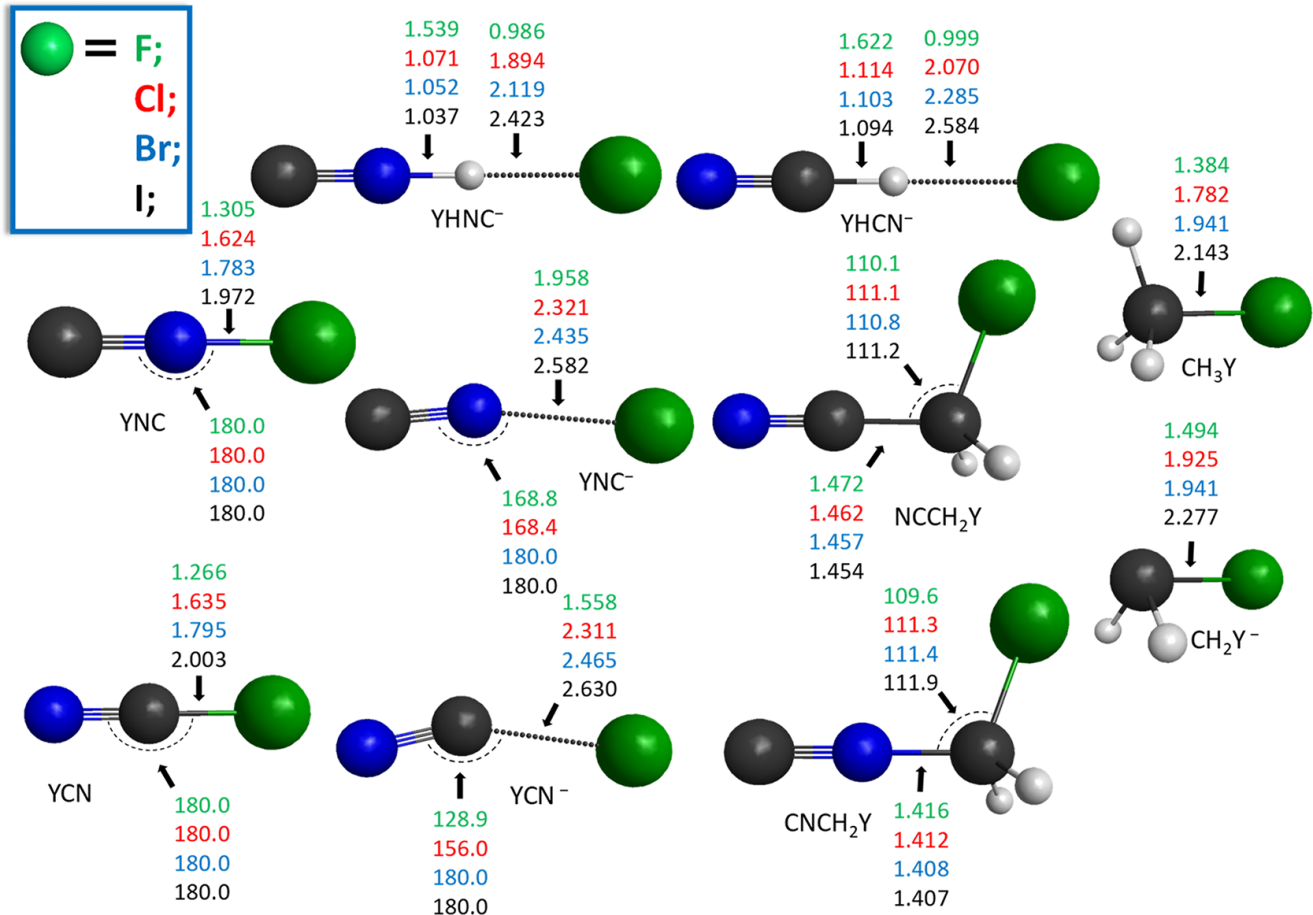
Benchmark equilibrium structures of the various halogen-containing products of the NC^–^/CN^–^ + CH_3_Y [Y = F, Cl, Br, and I] reactions showing the most important distances (Å) and angles (°) obtained at the CCSD(T)-F12b/aug-cc-pVTZ level of theory.

**Figure 6:**
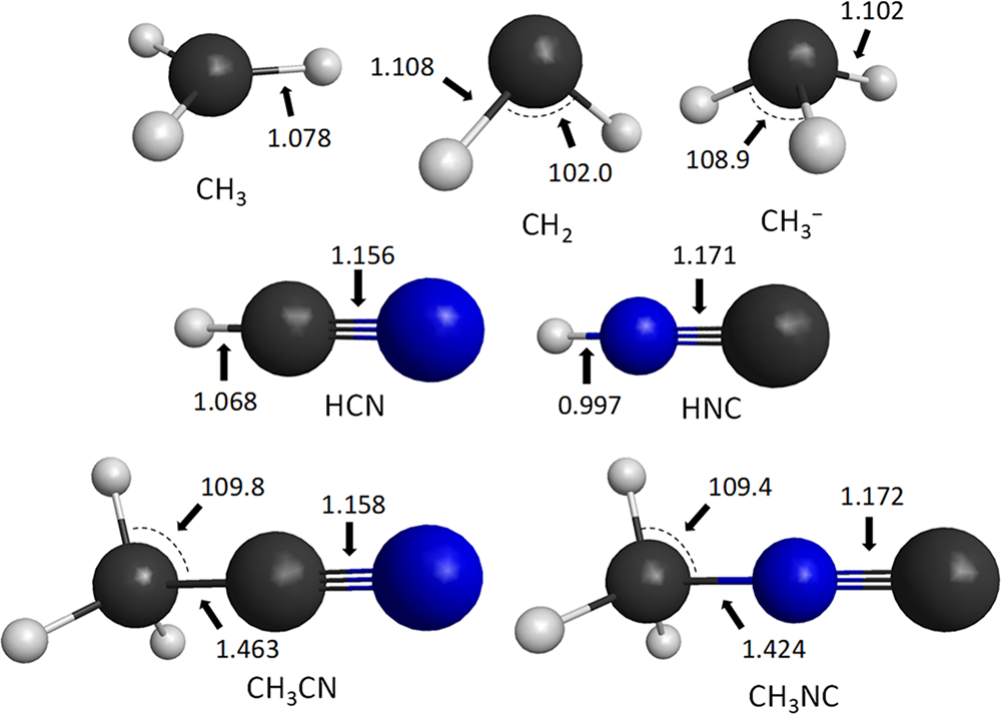
Benchmark equilibrium structures of the various non-halogen-containing products of the NC^–^/CN^–^ + CH_3_Y [Y = F, Cl, Br, and I] reactions showing the most important distances (Å) and angles (°) obtained at the CCSD(T)-F12b/aug-cc-pVTZ level of theory.

Besides the S_N_2 channels leading to Y^–^ + CH_3_CN/CH_3_NC, we consider other higher-energy product channels of the NC^–^/CN^–^ + CH_3_Y reactions such as proton abstraction (HCN/HNC + CH_2_Y^–^), hydride-ion substitution (H^–^ + YH_2_CCN/YH_2_CNC), halogen abstraction (YCN^–^/YNC^–^ + CH_3_ and YCN/YNC + CH_3_^–^), and YHCN^–^/YHNC^–^ complex formation (YHCN^–^/YHNC^–^ + ^1^CH_2_). The structures of the various products are shown in Figures 5 and 6, and the reaction enthalpies are given in Tables 1 and 2. The non-S_N_2 channels are always endothermic, the endothermicity decreases with the increasing atomic number of Y, and the C-bond formations are always favored thermodynamically. Usually, proton abstraction is the lowest-energy non-S_N_2 channel, with reaction enthalpies between 58.91–36.70 kcal/mol (HCN + CH_2_Y^–^) and 74.39–52.15 kcal/mol (HNC + CH_2_Y^–^), showing that the ZPE-corrected energy of HCN is below that of HNC by 15.5 kcal/mol. Halogen-abstraction forming two doublet products (YCN^–^/YNC^–^ + CH_3_) is found to be often competitive with the proton-abstraction channels, except for Y = F. For Y = Cl, Br, and I, the reaction enthalpies of the YCN^–^/YNC^–^ + CH_3_ channels differ by only a few kcal/mol from the enthalpies of the corresponding HCN/HNC + CH_2_Y^–^ products and for Y = Br and I, halogen abstraction is clearly less endothermic. Note that two doublet products like YCN^–^/YNC^–^ + CH_3_ can be formed on a singlet potential energy surface, and the singlet products (YCN/YNC + CH_3_^–^) have significantly higher energies as Tables 1 and 2 show, and thus, the latter products correspond to an excited electronic state. Considering the structures shown in Figure 5, one can see that YCN molecules are linear, whereas the YCN^–^ anions are bent for Y = F and Cl and linear for Y = Br and I. CH_3_ and CH_3_^–^ have planar and pyramidal structures with *D*_3h_ and *C*_3v_ point-group symmetry, respectively (Figure 6). The 0 K reaction enthalpies of hydride anion substitution are in the ranges of 57.08–58.10 and 75.96–80.67 kcal/mol for C–C and N–C bond formation, respectively, showing similar endothermicity as proton abstraction for Y = F, whereas hydride substitution is significantly more endotherm than proton abstraction for Y = Cl, Br, and I. The finding that the reaction enthalpies of hydride substitution do not show substantial Y dependence can be explained by the fact that the C–Y bond is a spectator in these processes and in every case, a C–H bond breaks heterolytically and a C–C/N–C bond forms, and thus the reaction enthalpies only depend significantly on the reactive site of the nucleophile. The reaction enthalpies of the YHCN^–^ + ^1^CH_2_ channels decrease from 66.40 to 51.65 kcal/mol with the increasing atomic number of Y, whereas for YHNC^–^ + ^1^CH_2_, the enthalpies are in a narrower range of 62–67 kcal/mol. These data are similar to those of hydride substitution in the case of YHCN^–^ formation, whereas they are significantly below the hydride substitution values for the YHNC^–^ channel. Here, two notes should be mentioned. First, the above results correspond to the singlet methylene (^1^CH_2_), whereas the ground electronic state of CH_2_ is triplet. We consider here ^1^CH_2_ because on a singlet potential energy surface, YHCN^–^/YHNC^–^ + ^1^CH_2_ can be formed, whereas triplet CH_2_ formation would proceed via non-adiabatic dynamics. Second, YHCN^–^/YHNC^–^ complexes are linear consisting of an YH and a CN^–^/NC^–^ fragment for Y = F and an Y^–^ and a HCN/HNC unit for Y = Cl, Br, and I, as the bond lengths show in Figure 5. This finding can be explained by considering the proton-affinity order of the Y^–^ and CN^–^/NC^–^ ions (F^–^ > NC^–^ > CN^–^ > Cl^–^ > Br^–^ > I^–^).

**Table 1:** Benchmark Classical and Adiabatic Energies with Auxiliary Energy Contributions Such as Post-CCSD(T), Core, Relativistic, and ZPE Corrections Relative to Reactants (in kcal/mol) for the Stationary Points and Different Product Channels of the NC^–^ + CH_3_Y [Y = F, Cl, Br, and I] Reactions

	MP2	CCSD(T)-F12b									
stationary points	aVDZ^a^	aVDZ^b^	aVTZ^c^	aVQZ^d^	δ[T]^e^	δ[(Q)]^f^	Δ_core_^g^	Δ_rel_^h^	classical^i^	Δ_ZPE_^j^	adiabatic^k^
NC^–^ + CH_3_F											
HMIN2	–10.12	–9.50	–9.45	–9.39	0.00	–0.03	0.01	0.00	–9.42	0.45	–8.97
PreMIN	–9.51	–8.99	–8.97	–8.88	–0.02	–0.03	0.00	0.00	–8.93	0.43	–8.51
WaldenTS	9.12	12.71	12.20	12.18	–0.12	–0.27	0.19	–0.04	11.94	0.17	12.11
PostHMIN2	–31.01	–25.12	–25.66	–25.72	0.00	–0.13	–0.54	0.05	–26.34	0.11	–26.24
FSTS	53.43	56.56	56.08	56.19	–0.15	–0.51	0.20	–0.04	55.69	–0.52	55.18
DITS	51.12	52.43	52.26	52.38	–0.08	–0.23	–0.25	0.03	51.85	–3.17	48.68
F^–^ + CH_3_CN	–7.28	–0.32	–1.12	–1.44	0.02	–0.15	–0.54	0.06	–2.06	0.63	–1.43
HCN + CH_2_F^–^	61.77	63.10	62.53	62.44	–0.14	–0.11	0.00	–0.01	62.18	–3.27	58.91
H^–^ + FH_2_CCN	62.14	60.30	60.96	61.20	0.14	–0.08	–0.37	0.00	60.89	–3.59	57.31
FCN^–^ + CH_3_	69.66	75.77	75.26	75.23	–0.28	–0.25	0.08	–0.05	74.74	–5.19	69.55
FCN + CH_3_^–^	80.44	79.70	79.47	79.45	0.01	–0.28	–0.22	0.00	78.96	–3.64	75.32
FHCN^–^ + CH_2_	73.88	72.89	72.83	72.99	–0.29	–0.04	0.42	–0.07	73.01	–6.61	66.40
NC^–^ + CH_3_Cl											
HMIN2	–11.18	–10.34	–10.47	–10.46	0.00	–0.04	0.02	0.02	–10.45	0.34	–10.11
PreMIN	–10.34	–9.64	–9.80	–9.75	0.76	–0.05	0.02	0.02	–9.01	0.32	–8.69
WaldenTS	–1.42	0.66	–0.05	–0.18	–0.11	–0.26	0.28	–0.04	–0.31	0.35	0.04
WaldenPostMIN	–51.94	–47.06	–47.85	–48.25	0.05	0.01	–0.36	0.11	–48.44	1.67	–46.77
PostHMIN2	–52.20	–47.26	–48.09	–48.44	0.05	–0.01	–0.38	0.11	–48.68	1.80	–46.89
FSMIN	1.01	0.54	0.72	0.80	0.01	–0.05	–0.02	–0.11	0.63	0.28	0.91
FSTS	47.12	47.76	47.11	47.04	–0.26	–0.65	0.28	–0.09	46.32	–0.56	45.76
DITS	37.84	38.72	38.47	38.45	0.00	–0.22	–0.26	0.12	38.09	–2.39	35.71
Cl^–^ + CH_3_CN	–37.83	–32.65	–33.67	–34.15	0.07	0.00	–0.32	0.11	–34.29	1.61	–32.68
HCN + CH_2_Cl^–^	49.25	50.05	49.11	48.92	–0.12	–0.11	0.11	0.02	48.82	–2.76	46.07
H^–^ + ClH_2_CCN	62.03	60.98	61.79	62.02	0.19	–0.11	–0.40	–0.03	61.68	–3.58	58.10
ClCN^–^ + CH_3_	49.90	54.49	54.02	53.90	–0.52	–0.15	0.17	–0.15	53.25	–4.47	48.78
ClCN + CH_3_^–^	72.22	72.69	72.96	72.94	0.04	–0.31	–0.20	0.00	72.48	–3.52	68.96
ClHCN^–^ + CH_2_	65.47	66.11	65.47	65.23	–0.33	0.07	0.44	–0.02	65.39	–6.03	59.36
NC^–^ + CH_3_Br											
HMIN1	–9.59	–9.38	–9.28	–9.22	–0.01	–0.04	–0.01	–0.05	–9.29	0.36	–8.93
HMIN2	–11.30	–10.86	–10.79	–10.77	0.00	–0.05	0.02	–0.08	–10.80	0.34	–10.46
PreMIN	–10.48	–10.21	–10.14	–10.09	–0.02	–0.06	0.01	–0.07	–10.16	0.32	–9.83
WaldenTS	–4.16	–3.73	–4.04	–4.17	–0.11	–0.25	0.25	–0.13	–4.28	0.43	–3.86
WaldenPostMIN	–57.05	–54.30	–54.72	–55.22	0.06	0.03	–0.21	0.04	–55.35	2.07	–53.28
PostHMIN2	–57.03	–54.22	–54.64	–55.10	0.05	0.02	–0.24	0.00	–55.27	2.15	–53.12
FSMIN	–3.44	–3.50	–3.48	–3.39	0.03	–0.13	–0.01	0.03	–3.51	0.34	–3.17
FSTS	43.29	42.23	41.79	41.67	–0.28	–0.67	0.29	–0.03	41.01	–0.39	40.62
DITS	35.34	36.45	36.30	36.30	0.01	–0.22	–0.13	–0.05	35.96	–2.30	33.66
Br^–^ + CH_3_CN	–43.95	–41.14	–41.71	–42.30	0.07	0.03	–0.07	0.15	–42.28	2.02	–40.26
HCN + CH_2_Br^–^	45.56	45.16	44.46	44.26	–0.14	–0.10	0.27	–0.04	44.29	–2.59	41.70
H^–^ + BrH_2_CCN	61.10	60.58	61.38	61.59	0.21	–0.12	–0.45	0.00	61.23	–3.59	57.64
BrCN^–^ + CH_3_	42.03	44.51	44.39	44.19	–0.45	–0.09	0.33	0.12	43.97	–4.31	39.67
BrCN + CH_3_^–^	70.95	73.08	73.06	73.00	0.06	–0.32	–0.05	0.05	72.70	–3.46	69.24
BrHCN^–^ + CH_2_	61.82	60.51	60.31	59.99	–0.32	0.09	0.53	–0.18	60.29	–5.36	54.93
NC^–^ + CH_3_I											
HMIN1	–10.06	–9.82	–9.65	–9.57	–0.01	–0.05	–0.06	–0.03	–9.70	0.51	–9.18
HMIN2	–11.34	–10.90	–10.82	–10.80	0.01	–0.05	–0.03	–0.03	–10.88	0.37	–10.52
HTS2	–9.99	–9.66	–9.58	–9.54	–0.01	–0.05	–0.05	–0.03	–9.65	0.39	–9.25
PreMIN	–10.50	–10.23	–10.13	–10.09	–0.03	–0.07	–0.04	–0.03	–10.23	0.29	–9.94
WaldenTS	–6.21	–5.77	–5.91	–6.07	–0.10	–0.26	0.18	–0.06	–6.25	0.46	–5.79
WaldenPostMIN	–62.10	–59.53	–59.99	–60.72	0.07	0.05	0.02	–0.04	–60.57	2.47	–58.09
PostHMIN2	–61.86	–59.20^l^	–59.65^l^	–60.31^l^	0.08^l^	0.03^l^	–0.06^l^	–0.05^l^	–60.25^l^	2.40^l^	–57.86^l^
FSMIN	–10.35	–10.15	–10.15	–10.13	0.06	–0.23	0.12	0.08	–10.19	0.30	–9.88
FSTS	40.43	38.95	38.55	38.35	–0.30	–0.74	0.35	–0.01	37.67	–0.31	37.35
DITS	31.51	32.26	31.97	31.92	0.03	–0.22	–0.07	–0.03	31.66	–2.04	29.62
I^–^ + CH_3_CN	–50.32	–47.72	–48.38	–49.21	0.09	0.06	0.28	–0.06	–48.79	2.42	–46.37
HCN + CH_2_I^–^	40.72	40.06	39.23	38.95	–0.13	–0.11	0.36	–0.09	39.07	–2.37	36.70
H^–^ + IH_2_CCN	60.18	59.91	60.76	60.99	0.22	–0.13	–0.50	0.04	60.58	–3.49	57.08
ICN^–^ + CH_3_	33.18	35.56	35.45	35.19	–0.32	–0.05	0.63	0.02	35.45	–3.77	31.67
ICN + CH_3_^–^	68.59	70.31	70.23	70.16	0.08	–0.33	0.19	–0.01	70.09	–3.31	66.78
IHCN^–^ + CH_2_	58.32	56.90	56.80	56.29	–0.31	0.12	0.73	–0.17	56.83	–5.18	51.65

a MP2/aug-cc-pVDZ.

b CCSD(T)-F12b/aug-cc-pVDZ.

c CCSD(T)-F12b/aug-cc-pVTZ.

d CCSD(T)-F12b/aug-cc-pVQZ relative energies at CCSD(T)-F12b/aug-cc-pVTZ geometries.

e [CCSDT – CCSD(T)]/aug-cc-pVDZ at CCSD(T)-F12b/aug-cc-pVTZ geometries.

f [CCSDT(Q) – CCSDT]/aug-cc-pVDZ at CCSD(T)-F12b/aug-cc-pVTZ geometries.

g Core correction obtained as the difference between AE and FC CCSD(T)/aug-cc-pwCVTZ energies at CCSD(T)-F12b/aug-cc-pVTZ geometries.

h Scalar relativistic effect obtained as DK-AE-CCSD(T)/aug-cc-pwCVTZ-DK – AE-CCSD(T)/aug-cc-pwCVTZ(-PP) [Y = F, Cl, and (Br and I)] at CCSD(T)-F12b/aug-cc-pVTZ geometries.

i Benchmark classical relative energies obtained as aVQZ + δ[T] + δ[(Q)] + Δ_core_ (+ Δ_rel_ for Y = F and Cl).

j ZPE corrections obtained at CCSD(T)-F12b/aug-cc-pVTZ.

k Benchmark adiabatic relative energies obtained as classical + Δ_ZPE_.

l MP2/aug-cc-pVDZ geometry and frequencies.

**Table 2:** Benchmark Classical and Adiabatic Energies with Auxiliary Energy Contributions Such as Post-CCSD(T), Core, Relativistic, and ZPE Corrections Relative to Reactants (in kcal/mol) for the Stationary Points and Different Product Channels of the CN^–^ + CH_3_Y [Y=F, Cl, Br, I] Reactions

	MP2	CCSD(T)-F12b									
stationary points	aVDZ^a^	aVDZ^b^	aVTZ^c^	aVQZ^d^	δ[T]^e^	δ[(Q)]^f^	Δ_core_^g^	Δ_rel_^h^	classical^i^	Δ_ZPE_^j^	adiabatic^k^
CN^–^ + CH_3_F											
HMIN2	–10.12	–9.48	–9.42	–9.37	0.00	–0.03	0.01	0.00	–9.39	0.40	–8.99
PreMIN	–10.00	–9.40	–9.40	–9.33	–0.01	–0.01	0.01	0.00	–9.34	0.39	–8.94
WaldenTS	14.67	18.36	17.85	17.81	–0.12	–0.21	0.23	–0.02	17.69	0.23	17.92
PostHMIN2	–4.08	–0.04	–0.36	–0.40	–0.09	–0.01	–0.31	0.08	–0.73	0.56	–0.18
FSTS	61.55	64.94	64.39	64.61	–0.20	–0.48	0.26	–0.03	64.16	–0.44	63.72
DITS	58.80	59.29	59.18	59.35	–0.10	–0.12	–0.08	0.04	59.09	–3.89	55.20
F^–^ + CH_3_NC	19.45	23.63	23.07	22.79	–0.11	0.08	–0.27	0.09	22.58	0.63	23.21
HNC + CH_2_F^–^	79.72	77.90	77.39	77.88	–0.30	0.18	0.19	0.02	77.98	–3.59	74.39
H^–^ + FH_2_CNC	83.59	78.30	79.17	79.44	0.03	0.21	–0.09	0.03	79.62	–3.66	75.96
FNC^–^ + CH_3_	99.84	97.24	97.59	97.73	–0.59	–0.47	0.17	–0.15	96.70	–5.38	91.31
FNC + CH_3_^–^	155.34	150.98	150.62	150.62	–0.20	–0.26	0.17	–0.05	150.28	–4.85	145.43
FHNC^–^ + CH_2_	74.77	73.18	73.11	73.21	–0.28	0.01	0.46	–0.06	73.34	–6.32	67.02
CN^–^ + CH_3_Cl											
HMIN2	–11.18	–10.32	–10.46	–10.45	0.00	–0.04	0.02	0.02	–10.44	0.33	–10.11
PreMIN	–10.80	–10.05	–10.18^l^	–10.15^l^	–0.01^l^	–0.02^l^	0.03^l^	0.02^l^	–10.15^l^	1.12^l^	–9.03^l^
WaldenTS	2.52	4.78	4.04	3.88	–0.11	–0.19	0.35	–0.03	3.90	0.40	4.29
WaldenPostMIN	–26.72	–23.91	–24.48	–24.85	–0.04	0.15	–0.11	0.14	–24.72	1.83	–22.89
FSMIN	0.56	0.18	0.52	0.62	0.00	–0.04	–0.02	–0.10	0.46	0.23	0.69
FSTS	52.41	53.47	52.75	52.72	–0.27	–0.60	0.37	–0.08	52.14	–0.28	51.87
DITS	47.06	46.78^m^	46.56^m^	46.60^m^	–0.04^m^	–0.07^m^	–0.08^m^	0.10^m^	46.51^m^	–2.74^m^	43.77^m^
Cl^–^ + CH_3_NC	–11.10	–8.71	–9.48	–9.91	–0.06	0.23	–0.04	0.15	–9.64	1.61	–8.03
HNC + CH_2_Cl^–^	67.20	64.85	63.96	64.37	–0.27	0.18	0.30	0.05	64.63	–3.08	61.55
H^–^ + ClH_2_CNC	86.54	82.02	83.22	83.51	0.07	0.17	–0.12	0.00	83.63	–3.70	79.93
ClNC^–^ + CH_3_	65.84	67.17	66.84	66.95	–0.36	–0.17	0.25	–0.21	66.46	–4.51	61.95
ClNC + CH_3_^–^	121.71	116.04	116.31	116.38	–0.13	–0.12	0.13	–0.03	116.23	–4.16	112.07
ClHNC^–^ + CH_2_	75.39	74.52	73.87	73.67	–0.38	0.19	0.54	0.01	74.03	–6.36	67.67
CN^–^ + CH_3_Br											
HMIN2	–11.32	–10.84	–10.78	–10.76	0.00	–0.05	0.02	–0.07	–10.78	0.34	–10.45
WaldenTS	–0.26	0.19	–0.15	–0.33	–0.10	–0.19	0.34	–0.12	–0.28	0.41	0.14
WaldenPostMIN	–31.67	–31.06	–31.25	–31.72	–0.04	0.18	0.03	0.06	–31.54	2.19	–29.35
FSMIN	–3.55	–3.18	–3.18	–3.08	0.01	–0.08	–0.01	0.03	–3.16	0.15	–3.01
FSTS	48.51	47.62	47.10	47.02	–0.27	–0.63	0.43	–0.04	46.55	–0.21	46.34
DITS	44.62	44.49	44.39	44.45	–0.02	–0.08	0.02	–0.03	44.36	–2.72	41.65
Br^–^ + CH_3_NC	–17.22	–17.20	–17.52	–18.07	–0.06	0.25	0.20	0.19	–17.67	2.02	–15.65
HNC + CH_2_Br^–^	63.51	59.96	59.31	59.71	–0.30	0.19	0.46	–0.01	60.06	–2.91	57.14
H^–^ + BrH_2_CNC	86.40	82.53	83.57	83.86	0.09	0.15	–0.19	0.05	83.91	–3.70	80.20
BrNC^–^ + CH_3_	54.17	55.37	55.31	55.21	–0.28	–0.10	0.46	0.16	55.29	–4.36	50.93
BrNC + CH_3_^–^	113.47	109.75	109.90	109.90	–0.11	–0.14	0.48	0.09	110.13	–4.03	106.10
BrHNC^–^ + CH_2_	73.23	70.12	69.83	69.57	–0.38	0.23	0.61	–0.16	70.03	–5.87	64.15
CN^–^ + CH_3_I											
HMIN2	–11.38	–10.89	–10.81	–10.80	0.01	–0.06	–0.04	–0.03	–10.88	0.33	–10.55
WaldenTS	–2.31	–1.94	–2.14	–2.35	–0.09	–0.21	0.31	–0.08	–2.33	0.51	–1.82
WaldenPostMIN	–36.54	–36.15	–36.40	–37.09	–0.02	0.21	0.26	–0.01	–36.64	2.59	–34.05
FSMIN	–9.45	–8.79	–8.79	–8.74	0.02	–0.13	0.05	0.08	–8.79	0.21	–8.58
FSTS	45.30	43.72	43.24	43.09	–0.24	–0.74	0.54	–0.03	42.65	–0.16	42.48
DITS	41.00	40.49	40.26	40.28	–0.01	–0.07	0.07	–0.01	40.27	–2.38	37.89
I^–^ + CH_3_NC	–23.60	–23.77	–24.19	–24.98	–0.04	0.28	0.55	–0.03	–24.18	2.42	–21.76
HNC + CH_2_I^–^	58.68	54.86	54.08	54.40	–0.29	0.18	0.55	–0.05	54.84	–2.69	52.15
H^–^ + IH_2_CNC	86.51	82.90	84.03	84.33	0.10	0.13	–0.25	0.08	84.32	–3.65	80.67
INC^–^ + CH_3_	42.06	44.20	43.96	43.84	–0.20	–0.02	0.81	0.05	44.43	–3.99	40.44
INC + CH_3_^–^	101.89	98.43	98.40	98.38	–0.11	–0.11	0.82	0.05	98.99	–3.81	95.17
IHNC^–^ + CH_2_	70.99	67.57	67.37	66.96	–0.38	0.28	0.78	–0.16	67.64	–5.39	62.25

a MP2/aug-cc-pVDZ.

b CCSD(T)-F12b/aug-cc-pVDZ.

c CCSD(T)-F12b/aug-cc-pVTZ.

d CCSD(T)-F12b/aug-cc-pVQZ relative energies at CCSD(T)-F12b/aug-cc-pVTZ geometries.

e [CCSDT – CCSD(T)]/aug-cc-pVDZ at CCSD(T)-F12b/aug-cc-pVTZ geometries.

f [CCSDT(Q) – CCSDT]/aug-cc-pVDZ at CCSD(T)-F12b/aug-cc-pVTZ geometries.

g Core correction obtained as the difference between AE and FC CCSD(T)/aug-cc-pwCVTZ energies at CCSD(T)-F12b/aug-cc-pVTZ geometries.

h Scalar relativistic effect obtained as DK-AE-CCSD(T)/aug-cc-pwCVTZ-DK – AE-CCSD(T)/aug-cc-pwCVTZ(-PP) [Y = F, Cl, and (Br and I)] at CCSD(T)-F12b/aug-cc-pVTZ geometries.

i Benchmark classical relative energies obtained as aVQZ + δ[T] + δ[(Q)] + Δ_core_ (+ Δ_rel_ for Y = F and Cl).

j ZPE corrections obtained at CCSD(T)-F12b/aug-cc-pVTZ.

k Benchmark adiabatic relative energies obtained as classical + Δ_ZPE_.

l CCSD(T)-F12b/aug-cc-pVDZ geometry and frequencies.

m MP2/aug-cc-pVDZ geometry and frequencies.

**Figure 7:**
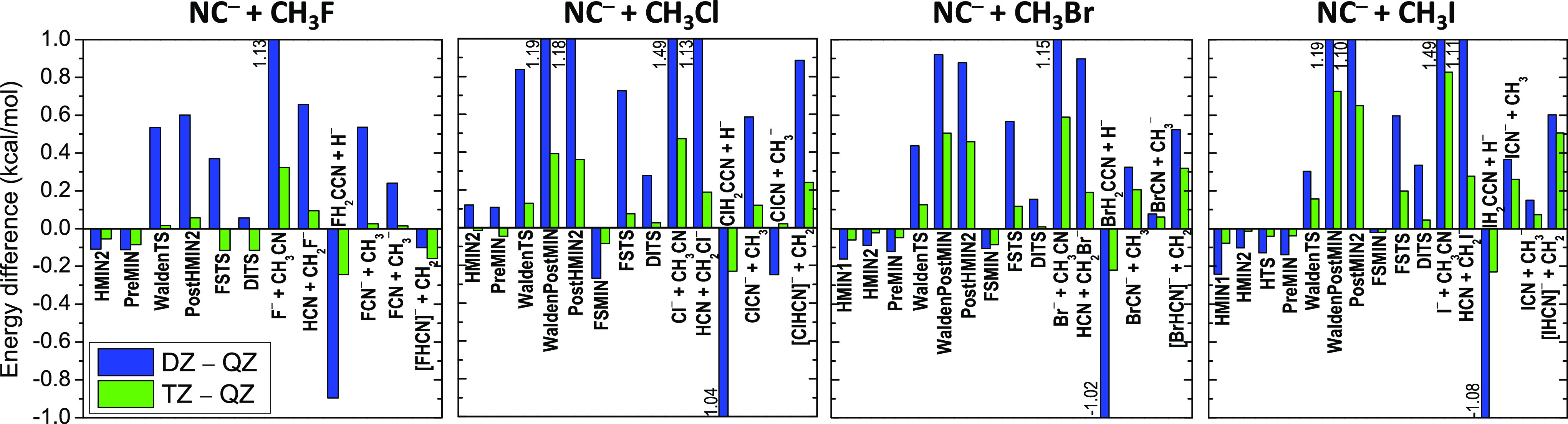
Convergence of the CCSD(T)-F12b relative energies for the stationary points and various product channels of the NC^–^ + CH_3_Y [Y = F, Cl, Br, and I] C-bond-forming reactions with the aug-cc-pVDZ (DZ), aug-cc-pVTZ (TZ), and aug-cc-pVQZ (QZ) basis sets.

**Figure 8:**
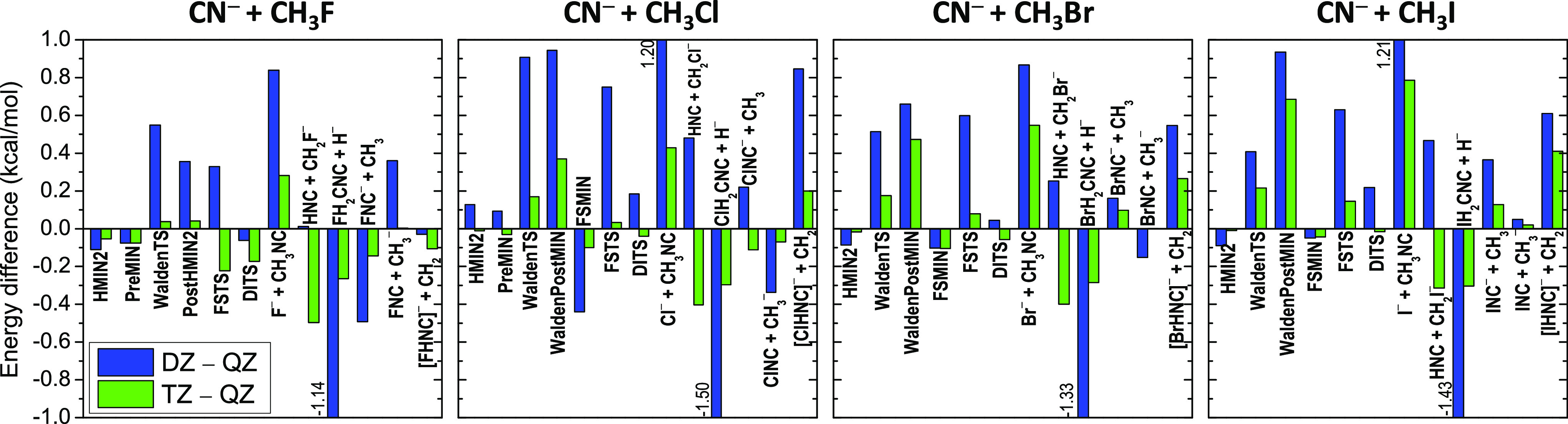
Convergence of the CCSD(T)-F12b relative energies for the stationary points and various product channels of the CN^–^ + CH_3_Y [Y = F, Cl, Br, and I] N-bond-forming reactions with the aug-cc-pVDZ (DZ), aug-cc-pVTZ (TZ), and aug-cc-pVQZ (QZ) basis sets.

**Figure 9:**
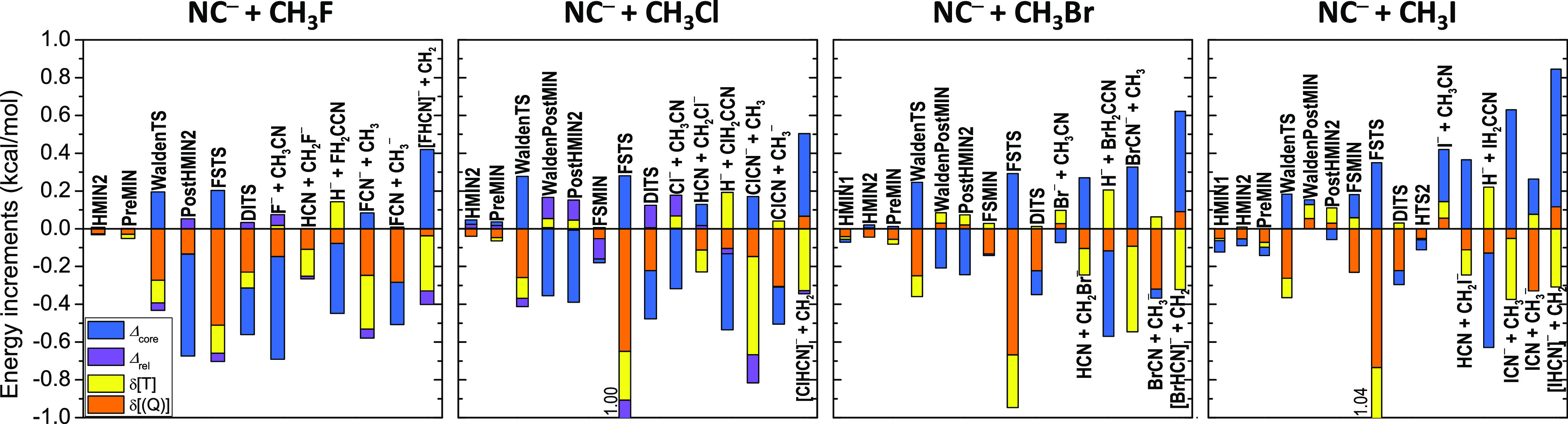
Core correlation (Δ_core_), relativistic (Δ_rel_), and post-CCSD(T) correlation (δ[T] and δ[(Q)]) corrections for the stationary points and various product channels of the NC^–^ + CH_3_Y [Y = F, Cl, Br, and I] C-bond-forming reactions. Δ_rel_ is not shown for Y = Br and I (DK – ECP results are given in Table 1).

**Figure 10:**
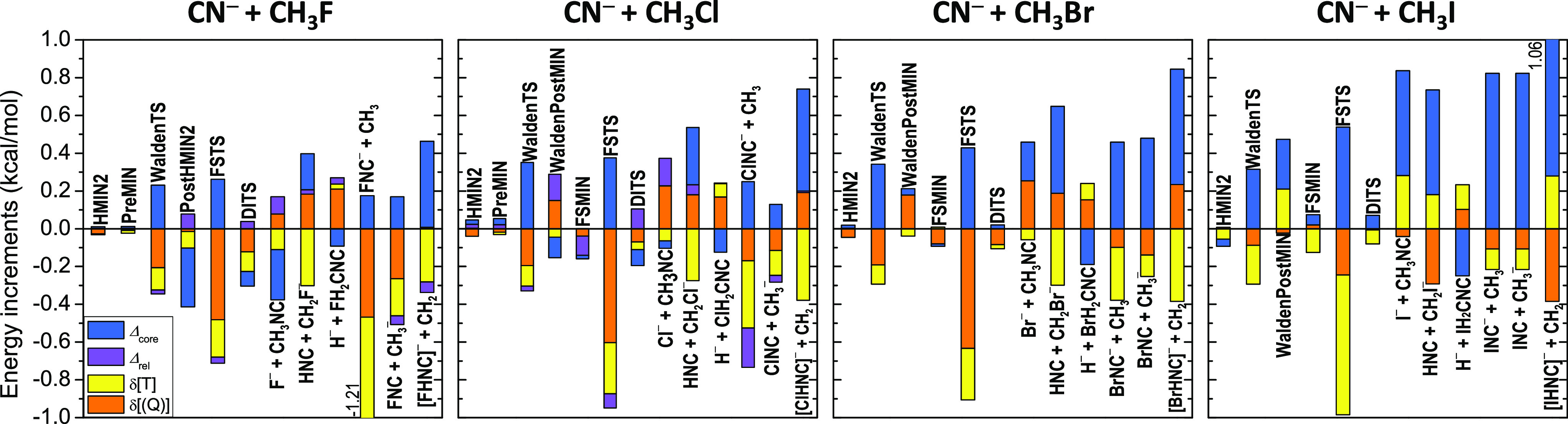
Core correlation (Δ_core_), relativistic (Δ_rel_), and post-CCSD(T) correlation (δ[T] and δ[(Q)]) corrections for the stationary points and various product channels of the CN^–^ + CH_3_Y [Y = F, Cl, Br, and I] N-bond-forming reactions. Δ_rel_ is not shown for Y = Br and I (DK – ECP results are given in Table 2).

Finally, we discuss the accuracy and uncertainty of the new benchmark energies considering the basis-set convergence and the magnitude of the different auxiliary corrections. The relative energies obtained by different ab initio levels of theory as well as the post-CCSD(T), core, relativistic, and ZPE corrections are given in Tables 1 and 2 for the title reactions with the C and N reactive site of the nucleophile, respectively. Graphical representations of the basis-set convergence of the CCSD(T)-F12b relative energies are shown in Figure 7 (C-bond formation) and Figure 8 (N-bond formation), and the core correlation, relativistic, and post-CCSD(T) correlation (δ[CCSDT] and δ[CCSDT(Q)]) contributions are depicted in Figure 9 (C-bond formation) and Figure 10 (N-bond formation). As Tables 1 and 2 show, the MP2 method performs reasonably well for the pre-reaction complexes since the MP2 and CCSD(T)-F12b relative energies usually agree within 0.5–1.0 kcal/mol. However, for the transition states and product channels, chemical accuracy is usually not achieved with the MP2 method, the absolute differences between the MP2 and CCSD(T)-F12b results are usually in the 1–5 kcal/mol range, but even larger deviations are also obtained. Thus, it is clear that the coupled-cluster method is needed to accurately account for the dynamical electron correlation in these systems. The explicitly correlated CCSD(T)-F12b method converges rapidly with the increasing size of the correlation-consistent basis sets as shown in Figures 7 and 8. Even with the aug-cc-pVDZ (DZ) basis set, most of the relative energies are basis-set converged within 1 kcal/mol. For the reactant-like structures (HMIN1, HMIN2, PreMIN, HTS, and FSMIN), the DZ results agree with the aug-cc-pVQZ (QZ) ones within about 0.1 kcal/mol. For the WaldenTS and FSTS, the DZ – QZ energy differences are larger, usually around 0.5 kcal/mol, and for DITS, the deviations are around 0.2 kcal/mol. Furthermore, in the case of the product-like structures and product channels, the DZ relative energies sometimes differ from the QZ results by more than 1 kcal/mol. Fortunately, increasing the basis set to aug-cc-pVTZ (TZ), these large deviations drop well below 1 kcal/mol and most of the TZ relative energies agree with the corresponding QZ data within 0.1–0.2 kcal/mol and the largest differences are around 0.5 kcal/mol. Based on these convergence tests, we may conclude that the QZ relative energies are usually basis-set converged within 0.1 kcal/mol. For more details about the accuracy of the QZ results and their comparison to the standard complete-basis-set-extrapolated energies, one may consult with ref (42) on Cl^–^ + CH_3_I. Considering the electron correlation beyond the gold-standard CCSD(T) level, we find that the δ[CCSDT] and δ[CCSDT(Q)] terms are usually ±(0.1–0.3) kcal/mol and often have the same sign, thus resulting in post-CCSD(T) correlation effects around ±(0.2–0.6) kcal/mol usually, but not always, with negative signs (Figures 9 and 10). The most substantial post-CCSD(T) corrections are obtained for the FSTSs (often around −1 kcal/mol, especially for Y = Cl, Br, and I) and for the FNC^–^ + CH_3_ channel (−1.06 kcal/mol). Core correlation corrections are usually negligible for the entrance-channel complexes but can be significant, ±(0.2–0.5) kcal/mol, for the transition states and product channels. The largest core correction values around 0.8 kcal/mol are obtained for the enthalpies of the CN^–^ + CH_3_I reaction, as somewhat expected. However, it is important to note that the magnitudes of the core correction values do not show significant Y dependence as Figures 9 and 10 show. Relativistic corrections are usually small (<0.1 kcal/mol) and have opposite signs than the corresponding, usually much larger, core corrections. The most substantial relativistic correction is −0.21 kcal/mol (ClNC^–^ + CH_3_). For the Y = Br and I systems, the Δ_rel_ values shown in Tables 1 and 2 correspond to the difference between DK and ECP results, where the latter already incorporates scalar relativity for the heavy halogen atoms. Therefore, these Δ_rel_ values are not included in our benchmark energies in the case of Y = Br and I; we rather use these data to estimate the uncertainty of the ECP computations. As seen in Tables 1 and 2, these DK – ECP values are usually less than 0.1 kcal/mol. Considering all the auxiliary corrections shown in Figures 9 and 10, we can conclude that the different contributions often partially cancel each other; however, in some cases, significant cumulative effects (>0.5 kcal/mol) still occur. Based on the above analysis of basis-set convergence and the magnitudes of the auxiliary corrections, we estimate that the uncertainty of our final benchmark classical relative energies is around 0.1–0.2 kcal/mol. To obtain the adiabatic results, the ZPE corrections have to be considered, which are given in Tables 1 and 2. As seen, Δ_ZPE_ is small, usually around 0.3–0.5 kcal/mol, for the pre-reaction complexes, WaldenTSs, and FSTSs, whereas it is significantly larger, 2–7 kcal/mol, for the DITSs and product channels. The ZPE corrections are positive for the pre-reaction complexes, WaldenTSs, and S_N_2 products, whereas they are negative for the other product channels, FSTSs, and DITSs. In some cases, especially for some of the product channels, the neglected anharmonicity (about 5% of Δ_ZPE_) may increase the uncertainty of the adiabatic relative energies. Thus, our prediction is that the present benchmark adiabatic relative energies are accurate within 0.1–0.4 kcal/mol.

## 4 Summary and Conclusions

Following our previous work^14^ on the C–C bond-forming NC^–^ + CH_3_Y [Y = F, Cl, Br, and I] S_N_2 reactions, in the present study, we have considered the ambident character of the nucleophile and characterized the stationary points for the N–C bond-forming pathways. Moreover, besides the S_N_2 channels, we have computed reaction enthalpies for various endothermic product channels such as proton abstraction, hydride-ion substitution, halogen abstraction, and YHCN^–^/YHNC^–^ complex formation. To obtain the best technically feasible ab initio properties of the stationary points, we have used the explicitly correlated CCSD(T)-F12b method with the aug-cc-pVTZ basis set to determine accurate structures and frequencies, and for energy computations, the basis set has been increased to aug-cc-pVQZ and auxiliary corrections have been computed such as post-CCSD(T), core, and relativistic corrections. The computations reveal that

(a) Thermodynamically, C–C bond formation is much more favored than N–C bond formation, whereas the kinetic preference of the former is less significant.
(b) Adiabatic barrier heights for Walden inversion are 12.1/17.9, 0.0/4.3, −3.9/0.1, and −5.8/–1.8 kcal/mol for C–C/N–C bond formation in the case of Y = F, Cl, Br, and I, respectively.
(c) Both double inversion and front-side attack proceed over high barriers in the range of 30–64 kcal/mol, the barrier heights decrease with the increasing atomic number of Y, and double inversion is always slightly more favored than front-side attack.
(d) Various ion-dipole, hydrogen-bonded, and halogen-bonded complexes are found in the entrance and/or product channels, which may play significant roles in the dynamics of the title reactions.
(e) All the non-S_N_2 product channels that can be obtained by adiabatic dynamics are endothermic with reaction enthalpies in the 31–92 kcal/mol range.
(f) The MP2 method may have a few kcal/mol uncertainty, CCSD(T)-F12b/aug-cc-pVQZ is basis-set converged within about 0.1 kcal/mol, post-CCSD(T) and core corrections can be around 0.5 kcal/mol, relativistic effects are usually negligible (<0.1 kcal/mol), and ZPE corrections can be a few kcal/mol. The estimated uncertainties of the new benchmark classical (adiabatic) relative energies are 0.1–0.2 (0.1–0.4) kcal/mol.

The present comprehensive ab initio stationary-point characterization of the title reactions is expected to guide future global potential energy surface developments and reaction dynamics studies, thereby revealing the competition between the above-proposed reaction pathways of an ambident nucleophile. Furthermore, future experiments may look for the different product ions formed by the various endothermic product channels investigated in the present work.
